# The scientific gap in reproductive immunology and the financial burden on patients: a critical review

**DOI:** 10.5935/1518-0557.20250191

**Published:** 2026

**Authors:** Pedro Augusto Araujo Monteleone

**Affiliations:** 1 Centro de Reprodução Humana Monteleone, São Paulo, Brazil

Reproductive immunology, a promising and biologically plausible field, has become an area where widespread clinical practices lack robust scientific validation. The uncritical adoption of interventions such as intravenous immunoglobulin (IVIG), corticosteroids, lymphocyte immunotherapy, and functional testing of uterine natural killer (NK) cells often escapes the methodological rigor required in evidence-based medicine.

While pharmaceutical products must undergo structured phases of clinical evaluation (from preclinical testing to post-marketing surveillance), and diagnostic tests are subject to analytical and clinical validation, these requirements are often relaxed in reproductive immunology. Interventions targeting implantation failure or recurrent pregnancy loss are commonly adopted based on retrospective observational studies or case series, frequently lacking control groups or blinding.

As Prof. Norbert Gleicher and colleagues (Gleicher *et al*., 2017) have emphasized, “reproductive medicine has become captive to biological plausibility at the expense of clinical evidence”. In multiple publications, these authors question not only the effectiveness of immunological interventions but also the ethical implications of their widespread clinical use in the absence of robust data, especially considering their cost and potential risks.

This distortion is jointly perpetuated by the pharmaceutical and diagnostics industries, which often avoid the significant investment required for clinical trials, opting instead to launch commercially attractive solutions based on preliminary findings. Private fertility clinics, in turn, implement these interventions under the guise of “personalized treatment,” shifting financial and clinical burdens onto patients, many of whom are emotionally vulnerable.

The absence of strong guidelines from regulatory agencies and medical societies exacerbates this situation, enabling the perpetuation of therapies with unproven efficacy for decades. A recent example is the article by Cavalcante *et al*. (2025), published in the Journal of Reproductive Immunology, which relied on a retrospective, uncontrolled design, with substantial selection bias and vague outcome definitions, illustrates how editorial standards may yield to commercial and institutional pressure.

Several tests and interventions that once gained broad popularity are now under scrutiny due to lack of reproducible benefit. The Endometrial Receptivity Analysis (ERA^®^) (Díaz-Gimeno *et al*., 2013), for instance, has failed to demonstrate consistent improvement in live birth rates, leading to its gradual decline in clinical use. The application of platelet-rich plasma (PRP) to the endometrium or ovaries also lacks validation from well-conducted randomized trials (Sfakianoudis *et al*., 2019). Intralipid therapy, although proposed by some as an immunomodulatory strategy, remains controversial regarding dosage, indication, and clinical outcomes.

It is imperative that the scientific community and regulatory bodies demand minimal standards of validation before new therapies are adopted clinically. To remain ethically responsible and scientifically sound, reproductive medicine must subject all interventions to the same level of methodological scrutiny expected in other fields. This is not a rejection of reproductive immunology-but rather, a call for it to be firmly grounded in evidence, not only in theoretical appeal.

Table 1 summarizes current guideline positions regarding key immunological interventions in assisted reproduction.

**Table 1:** Comparative Summary of Evidence and Guidelines.

Aspect	PRP Ovarian	NK in RPL	Guidelines ASRI	Biomarkers
ESHRE (2023)	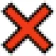	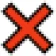	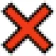	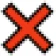
NICE (2017)	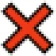	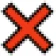	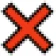	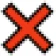
ASRI (Cavalcante *et al*., 2025)	-	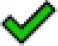	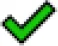	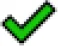

## References

[r1] Cavalcante MB, Harrity C, Luu T, Fatunbi J, Nakagawa K, Zhang Y, Zhang T, Alanazi H, Wu L, Lee S, Barini R, Kwak-Kim J. (2025). 2025 American Society for Reproductive Immunology Guidelines for
the Treatment of Recurrent Pregnancy Losses: Practice Recommendations From
the ASRI Clinical Reproductive Immunology Fellowship. Am J Reprod Immunol.

[r2] Díaz-Gimeno P, Ruiz-Alonso M, Blesa D, Bosch N, Martínez-Conejero JA, Alamá P, Garrido N, Pellicer A, Simón C. (2013). The accuracy and reproducibility of the endometrial receptivity
array is superior to histology as a diagnostic method for endometrial
receptivity. Fertil Steril.

[r3] Bender Atik R, Christiansen OB, Elson J, Kolte AM, Lewis S, Middeldorp S, Mcheik S, Peramo B, Quenby S, Nielsen HS, van der Hoorn ML, Vermeulen N, Goddijn M, ESHRE Guideline Group on RPL (2023). ESHRE guideline: recurrent pregnancy loss: an update in
2022. Hum Reprod Open.

[r4] Gleicher N, Kushnir VA, Barad DH. (2017). Redirecting reproductive immunology research toward pregnancy as
a period of temporary immune tolerance. J Assist Reprod Genet.

[r5] NICE: National Institute for Health and Care Excellence (2017). NICE guideline [NG156].

[r6] Sfakianoudis K, Simopoulou M, Nitsos N, Rapani A, Pantou A, Vaxevanoglou T, Kokkali G, Koutsilieris M, Pantos K. A (2019). Case Series on Platelet-Rich Plasma Revolutionary Management of
Poor Responder Patients. Gynecol Obstet Invest.

